# Acute Abdominal Pain Without Gross Hematuria as an Atypical Initial Presentation of Renal Arteriovenous Malformation

**DOI:** 10.7759/cureus.59367

**Published:** 2024-04-30

**Authors:** Kenichiro Iga, Yuji Okazaki, Kyungko Huh, Toshihisa Ichiba

**Affiliations:** 1 Emergency Department, Hiroshima City Hiroshima Citizens Hospital, Hiroshima, JPN

**Keywords:** renal arteriovenous malformation, gross hematuria, clot retention, acute abdominal pain, angiography

## Abstract

Congenital renal arteriovenous malformations (AVMs) occasionally manifest with recurrent gross hematuria, typically in young populations. Acute abdominal pain without previous episodes of gross hematuria in young women is frequently considered a diagnosis related to obstetric and gynecological conditions or acute appendicitis, excluding the possibility of clot retention, which is more commonly associated with the elderly. A 36-year-old woman with no history of gross hematuria presented with acute lower abdominal pain. Adnexal torsion was initially considered based on her symptoms and ultrasonography findings. However, contrast-enhanced computed tomography (CT) revealed clot retention and delayed contrast excretion in the right kidney. After bladder irrigation, she returned complaining of right flank pain. Subsequent plain CT revealed contrast pooling in the right kidney and hydronephrosis. In addition to these findings, small vessels in the right renal hilum were found to be prominent in the arterial phase on the first contrast-enhanced CT. Finally, angiography of renal arteries confirmed the diagnosis of a congenital cirsoid-type renal AVM, which was successfully treated with ethanol embolization. This case highlights the importance of understanding an atypical presentation of renal AVMs, which is acute abdominal pain, even in the absence of prior gross hematuria and the characteristic CT findings. Early diagnosis of renal AVMs is crucial for preventing potentially serious complications, including repeated clot retention and life-threatening rupture. The diverse clinical manifestations and images of renal AVMs should be recognized to facilitate prompt and accurate diagnosis.

## Introduction

Congenital renal arteriovenous malformations (AVMs) are characterized by abnormal connections between the renal arterial and venous systems through a vascular nidus, comprising a cluster of multiple, enlarged, tortuous arteriovenous communications [1]. The prevalence of renal AVMs in the general population is less than 0.04% [2]. This rare condition may be diagnosed incidentally or may occasionally manifest with recurrent gross hematuria, hypertension, and high-output heart failure between the ages of 20 and 40 years [1]. The primary complaint that would trigger a visit to a healthcare provider and a diagnosis of this condition would be gross hematuria [2]. Therefore, the early diagnosis of a renal AVM is difficult when a patient presents to the emergency department (ED) with symptoms other than the characteristic one. Acute lower abdominal pain in young women typically prompts consideration of ectopic pregnancy, adnexal torsion, ruptured ovarian cyst, and acute appendicitis [3]. In such cases, the possibility of clot retention, a condition that occurs predominantly in the elderly and is a urological emergency, may not be considered in the absence of prior gross hematuria [4]. We present a case of acute lower abdominal pain due to the first episode of clot retention caused by a congenital renal AVM in a young woman with no history of gross hematuria.

## Case presentation

A 36-year-old healthy female presented to our emergency department (ED) with a complaint of acute lower middle abdominal pain two hours after onset. One day before admission, she had dysuria without gross hematuria, but the symptom was spontaneously resolved. She had not experienced any traumatic event and was not taking any medications. On arrival, her vital signs were as follows: body temperature, 37.2 ℃; blood pressure, 181/147 mmHg; heart rate, 103 beats per minute; and oxygen saturation, 99% without supplementation of oxygen. Physical examination revealed lower abdominal distension and tenderness. Point-of-care ultrasonography of the abdomen and pelvis showed a quasi-circular and heterogeneous lesion with internal echo in the pelvis and no findings of hydronephrosis. Her white cell count was slightly increased, and her hemoglobin, platelet, and serum β-human chorionic gonadotropin levels were normal (Table 1).

**Table 1:** Laboratory examinations ALT, alanine transaminase; AST, aspartate transferase; γ-GTP, γ-glutamyl transpeptidase; ALP, alkaline phosphatase; LD, lactate dehydrogenase

Test	Hospital admission	Reference range
White blood counts (x 10^3^/μL)	11.1	3.3 - 8.6
Hemoglobin (g/dL)	11.6	11.6 - 14.8
Platelet (x 10^4^/μL)	31.9	15.8 - 34.8
AST (U/L)	16	13 - 30
ALT (U/L)	12	10 - 42
γ-GTP (U/L)	10	13 - 64
ALP (U/L)	40	38 - 113
LD (U/L)	180	124 - 222
Blood urea nitrogen (mg/dL)	11	8 - 20
Creatinine (mg/dL)	0.6	0.65 - 1.07
Creatinine kinase (U/L)	91	59 - 248
C-reactive protein (mg/dL)	0.09	< 0.14
β-human chorionic gonadotropin (U/L)	< 0.1	< 0.1

**Figure 1:**
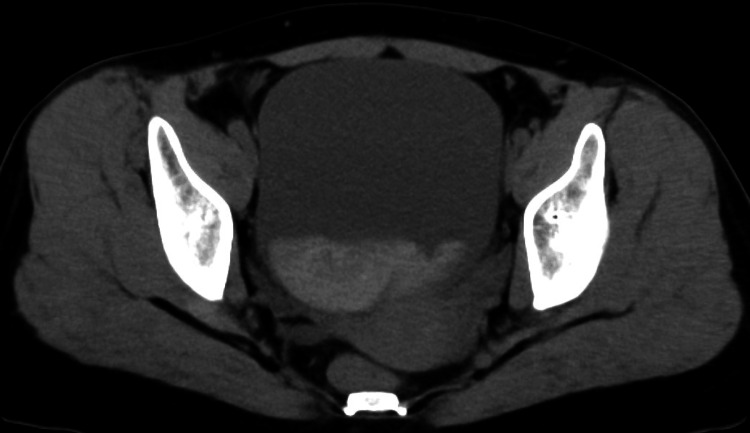
Computed tomography The initial contrast-enhanced computed tomography showed a distended urinary bladder containing hyperdense fluid, indicating clot retention.

**Figure 2:**
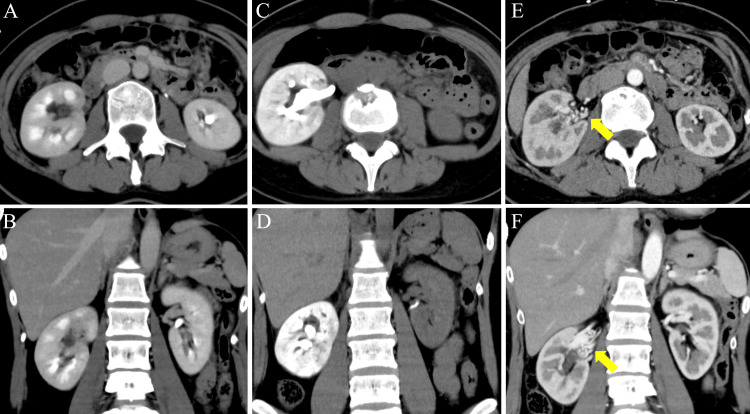
Computed tomography (A) Axial image. (B) Coronal image. The initial contrast-enhanced computed tomography (CT) in the equilibrium phase revealed delayed contrast excretion in the right kidney. (C) Axial image. (D) Coronal image. A subsequent plain CT demonstrated contrast pooling in the right kidney along with hydronephrosis. (E) Axial image. (F) Coronal image. The initial contrast-enhanced CT revealed small vessels in the right renal hilum, indicative of potential vascular malformations (yellow arrow).

Since adnexal torsion was suspected as a cause of acute abdominal pain, contrast-enhanced computed tomography (CECT) was performed. The CT showed a distended urinary bladder containing hyperdense fluid, indicating clot retention, and delayed contrast excretion in the right kidney (Figure 1 and Figures 2A, 2B).

**Figure 3:**
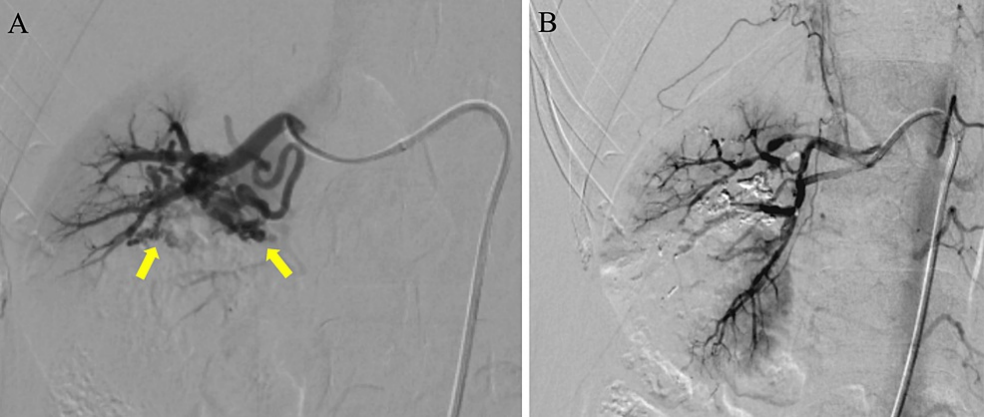
Angiography of the right renal artery (A) Angiography of the renal artery showed multiple shunts between arterioles and venules without a large venous sac, suggesting a cirsoid-type renal arteriovenous malformation (AVM) (yellow arrow). (B) Subsequent angiography showed resolution of the renal AVM after performing ethanol embolization.

Following a urological consultation, bladder irrigation for clot retention was carried out using a three-channel 22 Fr Foley catheter. Urinalysis showed no bacteriuria, and the emergency physician's reading of the contrast-enhanced CT showed that the etiology of the urinary retention was unknown. Seven hours post-discharge, she revisited our ED with acute-onset right flank pain with gross hematuria. A subsequent plain CT showed contrast pooling in the right kidney and hydronephrosis, consistent with upper urinary tract obstruction due to clots (Figures 2C, 2D). After the second CT, the radiologist observed that small vessels in the right renal hilum were pronounced on the first contrast-enhanced CT in the arterial phase, suggesting potential vascular malformations in the right kidney (Figures 2E, 2F). Considering the first CT findings, clot retention, and upper urinary tract obstruction due to clots, we suspected a renal AVM and proceeded with angiography of the right renal artery. The angiography confirmed the diagnosis of a congenital cirsoid-type renal AVM, and ethanol embolization was successfully performed (Figures 3A, 3B).

She was discharged without any complications and did not experience a recurrence of gross hematuria during a period of six months after treatment. Follow-up contrast-enhanced CT revealed resolution of the renal AVMs and an area of low density in the right renal parenchyma, suggesting a post-renal infarction change, and her kidney function did not worsen.

## Discussion

Renal AVMs may present with acute abdominal pain without gross hematuria before admission. This is due to clot retention caused by the formation of a clot, which is triggered by sudden-onset bleeding from the renal AVMs. Although physicians can easily suspect clot retention as a cause of acute abdominal pain if patients, even young patients, have recurrent gross hematuria, it is challenging to consider the possibility, especially in young patients with no episode of gross hematuria. Cases of repeated clot retention and life-threatening rupture of renal AVMs have been reported [5,6]. Thus, even in the first episode of clot retention, early diagnosis of renal AVMs would be desirable. Furthermore, in our case, the reason for not considering clot retention as the cause of acute abdomen was the absence of gross hematuria prior to admission, along with ultrasonographic findings suggestive of adnexal torsion. On abdominal ultrasound, the urinary bladder filled with clots may mimic a cystic pelvic lesion, potentially leading to a misdiagnosis of ovarian pathology [7]. Although clot retention can be easily diagnosed as a cause of acute abdominal pain through urinary drainage, it is crucial to recognize the diverse clinical manifestations of renal AVMs, beyond just repeated episodes of gross hematuria, in order to prevent serious complications arising from delayed diagnosis.

Clots resulting from a renal AVM can lead to obstructions not only in the lower urinary tract but also in the upper urinary tract. Upper urinary tract obstruction due to clots may present with symptoms similar to those of urolithiasis. In our patient’s second visit, we considered that patients presenting unilateral flank pain and gross hematuria are likely to be diagnosed with urolithiasis [8]; however, the initial presentation of clot retention suggested that the subsequent visit might be related to another clot episode. In addition, the delayed excretion of contrast material from the unilateral renal pelvis on the first CT in the equilibrium phase and the second CT strongly suggests obstruction in the ureter [9]. These characteristic CT findings may be useful in indicating that clot-related complications are due to bleeding from the kidney, which may be a key to suspecting renal AVMs.

In contrast, contrast-enhanced CT is useful for accurate diagnosis of renal AVMs, but diagnosing certain types can be challenging. Renal AVMs are categorized into types I to III based on their angiographic morphology [10]. Type I renal AVMs are direct arteriovenous fistulas featuring one or several distinct feeding arteries that directly shunt into a single draining vein with a large venous sac. Type II renal AVMs are numerous arterioles shunting to a single enlarged draining vein forming a large venous sac into which multiple feeders converge. Type III renal AVMs lack capillaries; instead, arteries and veins form shunts through abnormal vessels, creating an atypical vascular network without aneurysm formation. Clinicians should be aware of the potential difficulty in diagnosing renal AVMs using contrast-enhanced CT alone [5]. It is important to recognize that if a young patient presents with gross hematuria and the cause remains unclear after contrast-enhanced CT, renal artery angiography - the gold standard for diagnosis - may be considered to confirm a renal AVM.

Treatment of renal AVMs can be divided into non-surgical and surgical approaches. Non-surgical treatment typically involves endovascular treatment (EVT) with embolization [1]. This minimally invasive procedure uses catheters to deliver embolic agents such as autologous clots, polyvinyl alcohol, gelatin sponge, absolute ethanol, and n-butyl cyanoacrylate glue to occlude the abnormal blood vessels and disrupt the AVM. EVT with embolization is often the first-line treatment for symptomatic renal AVMs as in our case [1]. It can effectively treat the malformation while preserving the kidney. In contrast, surgical treatment is reserved for cases where embolization has failed (e.g., non-resolution of gross hematuria after EVT) or is not feasible, such as for large AVMs or those associated with malignancy [2]. The most definitive surgical option is total nephrectomy. Partial nephrectomy has been advocated as a nephron-sparing approach in select cases. Another surgical option is a ligation of feeding arteries in renal AVMs. However, these approaches result in renal parenchymal loss.

## Conclusions

Suspecting complications of renal AVMs can be challenging in cases without gross hematuria prior to admission. However, understanding the diverse clinical manifestations of renal AVMs is crucial for making an early diagnosis even in such atypical presentations. Furthermore, by fully grasping both the advantages and limitations of contrast-enhanced CT in diagnosing renal AVMs, the use of more invasive diagnostic tests, such as renal arteriography, can be facilitated when necessary. If not properly treated, renal AVMs can lead to severe complications. Therefore, prompt recognition and accurate diagnosis of this condition are essential for effective management and prevention of serious sequelae. This case report demonstrates the importance of suspecting renal AVMs in patients without gross hematuria, highlights the strengths and weaknesses of contrast-enhanced CT, and emphasizes the need for early diagnosis and appropriate treatment. By raising awareness of such atypical cases, it is hoped that the diagnosis and management of renal AVMs will be improved in the future.
